# Screening and genome analysis of heat-resistant and antioxidant lactic acid bacteria from Holstein cow milk

**DOI:** 10.3389/fmicb.2024.1455849

**Published:** 2024-11-14

**Authors:** Jiali Wang, Yunjiang Liu, Haohong Zheng, Jialiang Xin, Zhijun Zhong, Haifeng Liu, Yixin Huang, Hualin Fu, Ziyao Zhou, Guangneng Peng

**Affiliations:** Key Laboratory of Animal Disease and Human Health of Sichuan Province, College of Veterinary Medicine, Sichuan Agricultural University, Chengdu, China

**Keywords:** lactic acid bacteria, *Lactobacillus plantarum*, heat resistance, antioxidant capacity, heat stress, dairy cows

## Abstract

**Background:**

Heat stress significantly impacts dairy cows, primarily through oxidative stress, which undermines their health. The problem is exacerbated by the ongoing global warming trend. Lactic acid bacteria (LAB) are safe, economical, and readily accessible options for enhancing the host’s antioxidant defenses and preventing oxidative damage. They have been proven effective in alleviating heat stress-related damage, making them an excellent choice for protecting dairy cows from the adverse effects of heat stress.

**Method:**

In this study, five strains of LAB from Holstein cow milk (*Lactobacillus plantarum* L5, L14, L17, L19, L20) were evaluated for their heat resistance and antioxidant capacity by evaluating the growth characteristics and tolerance of the strains under high-temperature conditions, as well as their H_2_O_2_ tolerance, free radical scavenging ability (DPPH, OH^−^, ABTS), reducing ability, and EPS production ability. Furthermore, we employed Caco-2 cells to assess the adhesion rate of the strain, thereby confirming its ability to successfully colonize the host’s intestinal tract and ensuring the effective execution of its probiotic functions. The strain with excellent heat resistance and antioxidant capacity was then subjected to genomic analysis to gain insight into the molecular mechanisms behind their heat resistance, antioxidant capacity, and safety.

**Results:**

Among the two strains, *Lactobacillus plantarum* L19 emerges as a highly promising candidate. The strain exhibits robust growth even at high temperatures at 40°C and maintains a survival rate of 16.42% at the extreme temperature of 65°C. Furthermore, it demonstrates superior tolerance to hydrogen peroxide (27.3%), and possesses a notably higher free radical scavenging capacity with a high adhesion rate to Caco-2 cell (22.19%) compared to the other four strains tested. Genomic analysis revealed its’ genome has 17 genes related to antioxidants and three genes related to heat resistance. Importantly, L19 lacks any resistance genes, ensuring its safety as a probiotic.

**Conclusion:**

The results imply that *Lactobacillus plantarum* L19 has the potential to serve as an effective food additive in mitigating damages associated with heat stress. This research offers a valuable reference for the prevention and management of heat stress in dairy cows, while also expanding the scope of applications for LAB derived from cow milk.

## Introduction

Heat stress (HS) involves systemic adaptive responses to prolonged high temperatures, including non-specific defenses and specific physiological dysfunctions, occurring when thermoregulation is disrupted by extreme environmental heat (Peiris et al., 2017). Dairy cows are more prone to HS because of the high metabolic rate and small surface-to-volume ratio (Cartwright et al., 2021). Research indicates that HS not only leads to mastitis (Wang et al., 2021), liver metabolic (Fan et al., 2018), intestinal injury (Koch et al., 2019) and granulosa cell apoptosis in dairy cows (Wang et al., 2019), but also disrupts the immune system and causes reproductive disorders (Bagath et al., 2019) and decreased milk production (Bernabucci et al., 2014; Rhoads, 2023), seriously hindering the development of dairy farming industry. Due to global warming and rising temperatures, HS’s adverse effects will worsen. To the best of our knowledge, no safe and effective methods have been developed to prevent and control these effects in dairy cows. Experts suggest that without intervention, HS could directly cause losses of $40 billion by the end of this century (Thornton et al., 2022). Therefore, there is an urgent need to seek effective Intervention measures for HS damage to ensure the health of dairy cows and the prosperous development of the dairy farming industry.

Studies have shown that during HS, the energy metabolism and oxygen demand of body tissues increase, pushing mitochondrial activity beyond its thermal limit. This leads to mitochondrial damage and reactive oxygen species (ROS) release. The resulting overload of free radicals causes oxidative stress, which is responsible for tissue damage and cell apoptosis (Christen et al., 2018; Li et al., 2020; Liu et al., 2017). Therefore, boosting the antioxidant capacity in animals is a highly effective strategy for alleviating oxidative damage associated with HS (Li et al., 2024).

Probiotics are characterized as “live microorganisms that, when administered in sufficient quantities, confer health benefits to the host.” Lactic acid bacteria (LAB), a prominent group within the probiotic family, are naturally inhabitants of the gut flora. Notably, Lactobacillus species have been granted GRAS (Generally Recognized As Safe) status by the FDA (United States Food and Drug Administration) (Kelleher et al., 2017), making them a commonly used feed or food additive for both humans and animals, leveraging their advantageous effects. In recent years, a great many of research has highlighted the antioxidant potential of specific strains of LAB. These strains, along with their fermentation products, have demonstrated the ability to assist the host in resisting oxidative damage, thereby positioning them as high-quality natural antioxidants (Amaretti et al., 2013; Mishra et al., 2015). Therefore, *Lactobacillus* possesses the potential to prevent and control HS-related damage, a capability that has been validated by current research findings. In 2023, Cai’s research revealed that probiotics are capable of mitigating inflammation in male reproductive cells triggered by HS (Cai et al., 2023). Similarly, in 2014, Moore and colleagues explored the protective effects of *Bacillus subtilis* against HS-induced complications in rats, with their findings demonstrating that *Bacillus subtilis* can effectively alleviate the detrimental effects of HS (Moore et al., 2014). Unfortunately, there has been no report on the use of LAB to prevent and treat HS in dairy cows. Given that the beneficial properties of microorganisms are closely related to the particularity of the host, strains from the same host have stronger host adaptability and can better exert their beneficial properties (Jang et al., 2021; Zoetendal et al., 2006). In addition, high temperatures significantly affect the survival rate of probiotics, with most clinical probiotics requiring low-temperature storage to maintain their viability (Mahmoodian et al., 2024). During heat stress, the animal’s body temperature rises, potentially inactivating the probiotics and nullifying their beneficial effects. Consequently, strains that demonstrate robust tolerance to high temperatures exhibit more stable probiotic properties under heat stress, ensuring their efficacy in prevention and control strategies. Moreover, probiotics are frequently utilized as food additives, and they are often exposed to extreme temperatures. Heat-resistant strains not only withstand the production process’s high temperatures, enhancing strain utility and reducing costs but also facilitate clinical preservation, streamlining production and application (Matsushita et al., 2016). Therefore, screening cow-derived LAB with excellent heat resistance and antioxidant capacity is an promising approach method to alleviate HS oxidative damage for dairy cows.

In our previous work, we successfully isolated 26 strains of LAB from the milk of Holstein cows. Through a meticulous screening process, we identified five strains of *Lactobacillus plantarum* that exhibited resilience to acidic and bile conditions, possessed potent antibacterial properties and were non-hemolytic (*Lactobacillus plantarum* L5, L14, L17, L19, L20) (Zhang et al., 2022). The purpose of this study is to combine phenotypic and genomic analyses to select a strain with excellent heat resistance and antioxidant ability from the five LAB strains, as a candidate strain to alleviate heat stress-related damage, ensuring the health of dairy cows and the development of dairy farming.

## Materials and methods

### Source of LAB

The *Lactobacillus plantarum* strains L5, L14, L17, L19 and L20 are LAB with potential probiotic properties that were initially isolated from Holstein cow milk in our laboratory (Zhang et al., 2022).

### Preparation of cell-free supernatant and bacterial suspension

The LABs were activated for three generations and cultured overnight at 37°C in MRS liquid medium, centrifuged to collected the supernatant as cell-free supernatant (CFS) of LAB (8,000×*g* for 10 min at 4°C). The strain pellet was resuspended in sterile PBS to adjust the concentrations for bacterial suspension (BS) (1 × 10^8^ CFU/mL). In this study, all evaluations of LAB were performed by treating the strains according to the above method to obtain BS or CFS.

### High temperature resistance of LAB

#### Growth capacity at 40°C

The method was determined according to Liu et al. with some modifications (Liu et al., 2020). 50 μL BS of LAB was inoculation in 50 mL MRS broth (1%), cultured at 40°C under anaerobic conditions for 48 h. Measure the absorbance of the bacterial solution every 2 h from 0 to 24 h, every 4 h from 25 to 48 h (600 nm). Sterile MRS Medium was used as control group. Finally, draw the growth curve with times as the x-axis and OD_600_ as the y-axis.

#### Tolerance to high temperatures

The tolerance to high temperatures of LAB was assessed according to the method described by Da and Zhao with some modifications (Da et al., 2023; Zhao et al., 2023). 50 μL BS was inoculation in 50 mL of MRS broth (1%), and the inoculated culture was subjected to different temperature conditions for 30 min at 45°C, 55°C, and 65°C, respectively. Following this, the cultures were incubated at 37°C under anaerobic conditions for a period of 8 h. Upon completion of the 8 h incubation, the optical density of the experimental solutions was measured at 600 nm. Concurrently, BS inoculation in MRS broth without prior temperature treatment as control. Calculate the survival rate of each strain at different temperatures according to the following formula: Survival rate (%) = (experimental OD_600_/control OD_600_) × 100.

### Antioxidative ability

#### Resistance to hydrogen peroxide (H_2_O_2_)

The method of Li et al. was used with some modifications (Li et al., 2012). Prepare the BS according to the method described above. BS with a volume ratio of 2% was inoculated into MRS liquid medium supplemented with varying concentrations of H_2_O_2_: 0, 0.5, 1.0, 1.5 and 2.0 mmol/L. The inoculated cultures were then incubated at 37°C under anaerobic conditions for 8 h, then measured the OD_600_ of the culture. The BS inoculated into MRS liquid medium without H_2_O_2_ as the control group. Resistance rate (%) = (OD_experimental_/OD_control_) × 100.

#### 1, 1-diphenyl-2-picrylhydrazyl (DPPH) free radical scavenging ability

According to the method reported by Wang et al., to measure the DPPH free radical scavenging ability of LAB (Wang et al., 2023). Specifically, Prepare the CFS and BS of LAB using the method described above. Then, 2 mL 0.2 mmol/L DPPH anhydrous ethanol solution was mixed with 1 mL CFS or BS. React in the dark at room temperature for 30 min, then collect the supernatant (8,000×*g* for 10 min at 4°C). The OD_517_ of the supernatant was measured as test groups’ results (OD_test_). Concurrently, a blank group and a control group were established for comparative analysis. In the blank group, anhydrous ethanol was substituted for DPPH; while in the control group, sterile distilled water replaced both the BS and the CFS. The same determination methods were then replicated to acquire the test results for the blank group (OD_blank_) and the control group (OD_control_), respectively. The DPPH free radical clearance rate (%) was defined as: [1 − (OD_test_ − OD_blank_)/OD_control_] × 100.

#### Hydroxyl (OH^−^) radical scavenging ability

The OH^−^ radical scavenging assay was referred to Alam’s report (Alam et al., 2013). Add 1 mL ortho-phenanthroline (0.1%), 1 mL PBS, 1 mL FeSO_4_ (2.5 mmol/L), and 1 mL H_2_O_2_ (20 mmol/L) to 0.5 mL of CFS or BS of LAB, culturing in 37°C under anaerobic conditions for 1.5 h. Then measured the OD_536_ of the reaction solution (OD_test_). At the same time, anhydrous ethanol was used to replace the same volume of H_2_O_2_ as the blank group; distilled water was used to replace the same volume of sample solution as the control group. Repeat the above test method to obtain the results of the blank group (OD_blank_) and the control group (OD_control_). The OH^−^ scavenging activity rate (%) was expressed as: [1 − (OD_test_ − OD_blank_)/OD_control_] × 100.

#### 3-ethylbenzthiazoline-6-sulfonic acid (ABTS) free radical scavenging ability

The ability to scavenge ABTS free radicals was measured according to Prete et al. (Prete et al., 2020). Mix ABTS stock solution (7 mM) with 2.45 mM potassium persulfate to generate ABTS radical cation (ABTS.+), and store the mixture in the dark for 16 h at room temperature. Before use, adjust the ABTS.+ to 0.9 ± 0.0.02 at OD_734_ nm using PBS as the working solution. Next, add 0.25 mL BS or PBS (as control) to 1 mL working solution prepared above. Allow the mixture to react for 5 min, followed by centrifugation (4,000×*g* for 5 min at 4°C). Subsequently, detect the absorbance of the supernatant at OD_734_ nm. The ABTS free radical scavenging ability (%) was defined as the following equation: (1 − OD_test_/OD_control_) × 100.

#### The ability of reducing

Referring to the method of Kuda et al. (Kuda et al., 2009). Mixed 500 μL CFS or BS with 500 μL potassium ferricyanide (1%) and 500 μL PBS (pH = 6.6), reacting at 50°C water bath for 20 min. Waiting for cooling and adding 500 μL trichloride acetic acid (TAC, 10%). After centrifugation (8,000×*g* for 10 min at 4°C), 1 mL of supernatant was mixed with 1 mL ferric chloride (0.1%) and reacting at room temperature for 10 min, then measured OD_700_ of the resulting solution.

#### Exopolysaccharide (EPS) generation capacity

The ability to produce EPS was evaluated regarding the method of Wang et al. (Wang et al., 2023). The CFS of LAB was mixed with 800 mg/mL trichloroacetic acid (TCA) to get a final concentration of 40 mg/mL. The mixture was then incubated at 4°C overnight and centrifuged to collect the supernatant (8,000×*g* for 10 min at 4°C). Then, 250 μL of the supernatant was mixed with 250 μL phenol solution (6%) and 1 mL concentrated sulfuric acid on ice. Finally, OD_490_ of the resulting solution was measured. The standard curve was made by Glucose with different concentrations (3.125 mg/L, 6.25 mg/L, 12.5 mg/L, 25 mg/L, 50 mg/L, and 100 mg/L) to calculate the concentration of EPS.

#### Adhesion to Caco-2 cell line

According to the method in the report with minor modifications (Foongsawat et al., 2023). Caco-2 cells were seeded into a 12-well plate (1 × 10^5^/well) and cultured in the DMEM medium containing 10% FBS to achieve a confluent monolayer cell. Then, washed the cells’ monolayer thrice with PBS, and 1 mL of BS with a concentration of 1 × 10^8^ CFU/mL (C_0_) in DMEM medium was added to each well in three replicates. The plates were incubated at 37°C with 5% CO_2_ for 2 h. After incubation, aspirated the bacterial suspension and washed the cells monolayer thrice with PBS to remove non-adherent bacteria. Subsequently, the cells were digested with 100 μL trypsin (0.25%). The collected digested cell liquid yielded a suspension of colonies adherent to the cells. This suspension was then plated onto MRS solid medium for colony enumeration, allowing for the calculation of the number of colonies adhering to the cells (C_1_). Calculate the adhesion rate (%) according to the formula: Adhesion rate (%) = C_1_/C_0_ × 100.

### Whole-genome analyses

#### DNA extraction, sequencing

The genomic DNA of the candidate strain was extracted using a bacterial genomic DNA extraction kit (Tiangen, Beijing, China), then the purified DNA was submitted to Nanjing Paison Gene Technology Co., Ltd. for simultaneous sequencing using the second-generation platform Illumina NovaSeq and the third-generation platform Oxford Nanopore ONT.

Using Adapter Removal software to remove adapter contamination and SOAPec software for quality correction to obtain high-quality second-generation sequencing data (Schubert et al., 2016); use HGAP and CANU software to splice third-generation offline data to obtain counting sequences. Finally, Pilon software was used to correct the data to obtain the complete genome sequence (Walker et al., 2014).

#### Genome annotation

Transfer RNA (tRNA) were identified using the tRNA scan-SE, ribosomal RNA (rRNA) was determined using the Barrnapand other non-coding RNAs (ncRNAs) were predicted by comparing with the Rfam database (Kalvari et al. 2018).

The clustered regularly interspaced short palindromic repeat sequences (CRISPR) finder was used to determine CRISPR in the LAB (Grissa et al., 2007). The coding genes were annotation by Clusters of Orthologous Groups (COG), Kyoto Encyclopedia of Genes and Genomes (KEGG) (Moriya et al., 2007) and Gene Ontology (GO) (Conesa and Götz, 2008). Finally, the genome sequence, prediction information, and functional annotation information were integrated into a standard GBK (Genbank) format file, and the genome circle map was drawn using CGview software (Stothard and Wishart, 2005).

#### Bioinformatics analyses

To further elucidate the heat resistance and antioxidant capabilities of the strain at the molecular level, the heat resistance genes and antioxidant-related genes of the strain were mined. In addition, the antibiotic-resistance genes (AMR) were identified using ResFinder 4.0 with a 90% identity threshold and 60% minimum coverage.

#### Nucleotide sequence accession numbers

The genome of the candidate strain (*Lactobacillus plantarum* L19) was submitted and deposited in the NCBI database, accession numbers: SRR28342006.

### Statistical analysis

The measurements were independently replicated three times, and the findings were shown as the mean ± standard deviation by analysis in SPSS 27.0 (SPSS Inc., Chicago, USA). Statistical analyses were conducted using GraphPad Prism version 9.5 for Windows (GraphPad Software, San Diego, CA, USA).

## Results

### Growth capability under typical HS temperature

**Figure 1:**
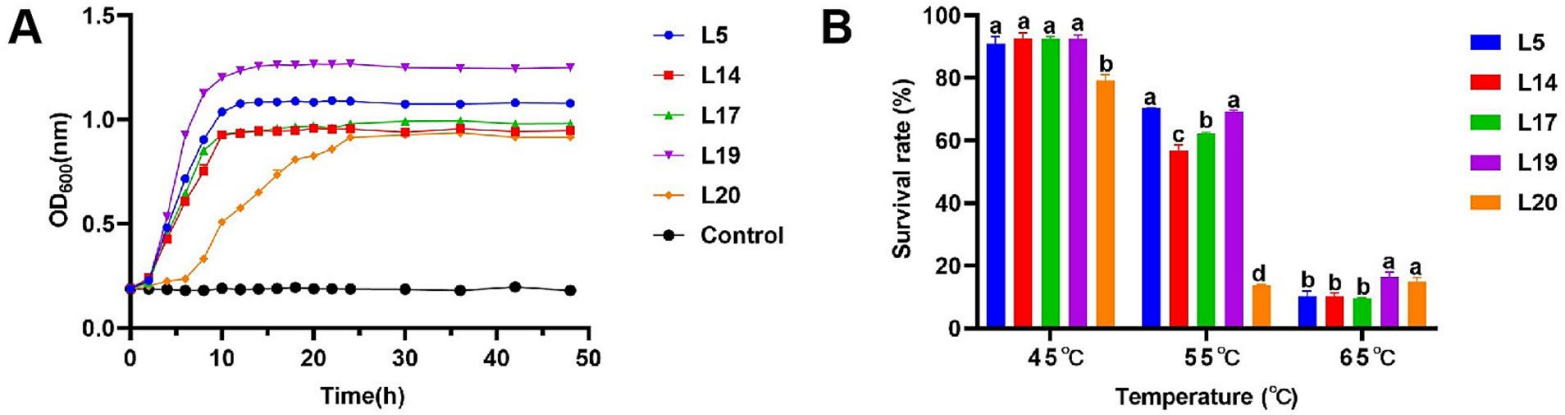
High temperature resistance of LAB. **(A)** Growth curve of LAB at 40°C; **(B)** Survival rate in different high temperature of LAB. The data are expressed as mean ± SD; Different letters represent significant difference (*p* < 0.05).

Under typical HS temperature conditions (40°C), *Lactobacillus plantarum* (L5, L14, L17 and L19) entered the logarithmic growth phase after a lag period of 0–2 h and reached a stabilizer in about 10 h, showing excellent growth characteristics. However, *Lactobacillus plantarum* L20 entering the logarithmic growth phase after about 6 h and entering the stable phase after 16 h, showing relatively poor growth ability compared with the other four strains. Among the strains tested, L5 and L19 demonstrated robust proliferation capabilities, as evidenced by their OD_600_ values surpassing 1.0 upon reaching the plateau phase of growth. In contrast, the OD_600_ values for the remaining three strains did not rise above 1.0 throughout their entire growth cycles (Figure 1A).

### High temperature resistance of LAB

The heat resistance test revealed that as temperatures rose, the survival rates of the five bacterial strains decreased. At 45°C, their survival rates ranged from 79.25 to 92.62%. Notably, L20 (79.25%) exhibited the lowest survival rate, while L14 (92.64%) demonstrated the highest survival rate. L5 (90.91%), L17 (92.44%), L19 (92.36%), and L14 (92.64%) all exhibited commendable tolerance with no significant differences observed in their survival rates. At 55°C, survival rates dropped to between 13.88 and 70.39%. Among the five strains, L5 (70.39%) and L19 (69.22%) exhibited notably higher survival rates, which were significantly greater than the other three strains. At 65°C, the survival rates of the five strains ranged from 9.62 to 16.42%, with L19 (16.42%) showing the highest survival rate, followed by L20 (14.92%), L5 (10.26%), L14 (10.16%), L17 (9.62%). Analyzing the survival rate data across different temperatures, L5 and L19 exhibited relatively robust survival rates under various high-temperature conditions, indicating a more stable heat resistance in these strains (Figure 1B).

### Antioxidative ability

#### Tolerance to H_2_O_2_ of LAB

The tolerance results of LAB to H_2_O_2_ are shown in Table 1. At concentrations of 0.5 mol/L H_2_O_2_, L5 (96.04%), and L19 (99.05%) exhibit strong tolerance, with their survival rates significantly surpassing those of the other three strains. When the H_2_O_2_ concentration is raised to 1 mmol/L, L19 (94.36%), L17 (92.15%), and L5 (80.89%) emerge as the most tolerant strains. At a concentration of 1.5 mmol/L, L5 (48.67%) demonstrates a particularly robust tolerance, significantly outperforming the other four strains. Finally, at an H_2_O_2_ concentration of 2 mmol/L, L19 (27.30%) showed the highest survival rate. Considering the survival rates of the strains across various concentrations, it is evident that L5, L17, and L19 exhibit relatively high tolerance to H_2_O_2_.

**Table 1:** The survival rate of LAB to different concentrations of H_2_O_2_.

Strain	Survival rate (%)			
	0.5 mmol/L	1 mmol/L	1.5 mmol/L	2 mmol/L
L5	96.04 ± 0.01a	80.89 ± 0.02b	48.67 ± 0.01a	15.13 ± 0c
L14	89.85 ± 0.01b	74.88 ± 0.01c	14.77 ± 0d	9.74 ± 0.01e
L17	85.20 ± 0.02b	92.15 ± 0.02a	25.95 ± 0.03c	18.44 ± 0b
L19	99.05 ± 0a	94.36 ± 0.03a	43.13 ± 0.01b	27.30 ± 0.01a
L20	75.18 ± 0.04c	42.23 ± 0.02d	27.48 ± 0.03c	11.77 ± 0.01d

The data are expressed as mean ± SD. Different letters represent significant difference (*p* < 0.05).

#### The scavenging ability of LAB to DPPH and OH^−^ free radicals

The results of the DPPH and OH^−^ free radical scavenging ability of the five strains are shown in Table 2. Among them, the CFS of L19 had the highest scavenging rate to DPPH (92.92%), followed by the CFS of L5 (90.86%) and L14 (89.71%). In addition, the CFS of L5 showed the highest ability to clear OH^−^, reaching 88.43%, which was significantly different from that of other strains (*p* < 0.05). The CFS of L19 (83.97%) and L14 (79.92%) followed in OH^−^ scavenging capacity. In conclusion, L5, L14, and L19 strains exhibited robust abilities in scavenging both DPPH and OH^−^ free radicals.

**Table 2:** The DPPH and OH^−^ radical scavenging rate of LAB.

Strain	DPPH (%)		OH^−^ (%)	
	Cell-free supernatant	Bacterial suspension	Cell-free supernatant	Bacterial suspension
L5	90.86 ± 0.11b	16.81 ± 0.07b	88.43 ± 0.84a	40.43 ± 1.55b
L14	89.71 ± 0.09b	11.91 ± 0.48d	79.92 ± 0.68c	38.17 ± 0.36c
L17	87.27 ± 0.77c	11.84 ± 0.21d	77.98 ± 0.66d	29.1 ± 0.87d
L19	92.92 ± 0.28a	27.60 ± 0.35a	83.97 ± 1.39b	46.95 ± 0.72a
L20	88.03 ± 1.36c	14.98 ± 0.36c	73.96 ± 0.88e	29.3 ± 1.36d

The data are expressed as mean ± SD. Different letters represent significant difference (*p* < 0.05).

#### The ability to scavenge ABTS radicals

**Figure 2:**
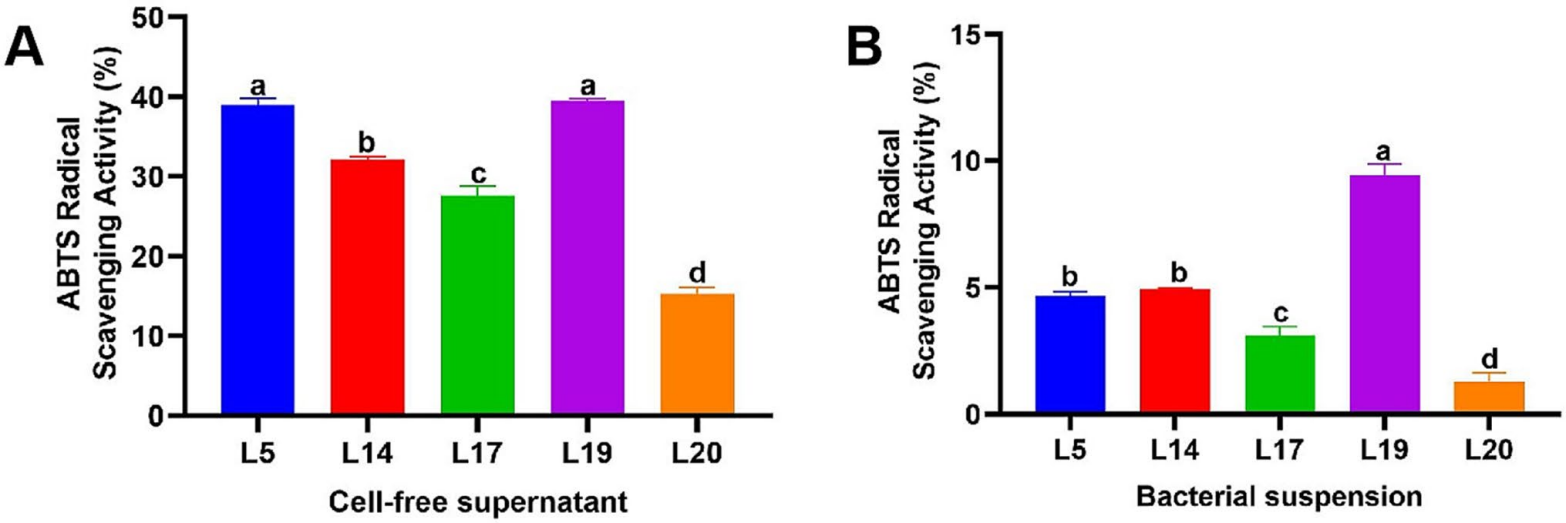
ABTS free radical scavenging rate of LAB. **(A)** ABTS free radical scavenging ability of the CFS; **(B)** ABTS free radical scavenging ability of the BS. The data are expressed as mean ± SD. Different letters represent significant difference (*p* < 0.05).

Figure 2 illustrates the ABTS scavenging capacity of the CFS and BS of LAB. Notably, the antioxidant capabilities of the CFS across all strains are markedly superior to those of the BS. The scavenging efficacy of the CFS across the five strains varied from 15.30 to 39.53%, while the BS demonstrated a less pronounced range, from 1.31 to 9.46%. Specifically, the CFS from strains L5 (39.04%) and L19 (39.53%) exhibited a particularly high ABTS scavenging ability, especially L19 which significantly differs from the other three strains (*p* < 0.05). Conversely, L20 exhibited the lowest scavenging rates in CFS (15.30%) and BS (1.31%).

#### Reducing ability

Table 3 presents the results of the reducing capacity of LAB. It is evident that the reducing ability of the CFS from all five strains surpasses that of the bacterial BS. Among these, the CFS derived from L5 exhibited the most superior reducing ability, this was closely followed by the CFS of strains L19 and L14, which also exhibited considerable reducing properties. In terms of the BS, the reduction analysis revealed that L19 had the most potent reducing ability among the five strains, significantly outperforming the other four LAB strains in this regard (*p* < 0.05).

**Table 3:** The reducing ability of LAB.

Strain	Reducing ability (OD_700_)	
	Cell-free supernatant	Bacterial suspension
L5	0.74 ± 0.05a	0.08 ± 0.00e
L14	0.58 ± 0.04bc	0.12 ± 0.00b
L17	0.55 ± 0.04bc	0.1 ± 0.00c
L19	0.62 ± 0.02b	0.13 ± 0.00a
L20	0.5 ± 0.06c	0.09 ± 0.00d

The data are expressed as mean ± SD. Different letters represent significant difference (*p* < 0.05).

#### EPS production

**Figure 3:**
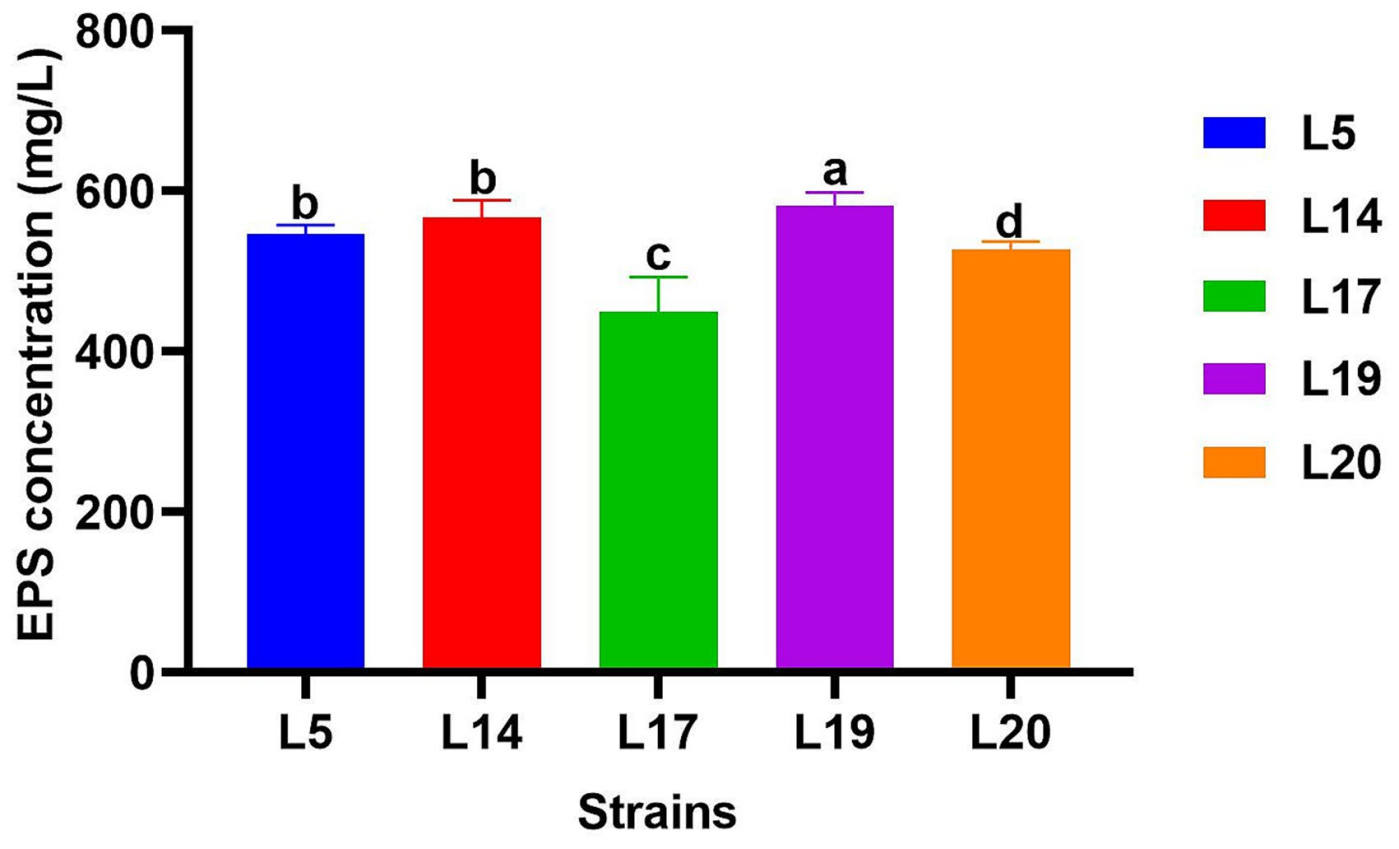
EPS production of LAB. The data are expressed as mean ± SD. Different letters represent significant difference (*p* < 0.05).

Figure 3 displays the EPS production capabilities of the five strains. All five strains possess the ability to produce EPS, with strain L19 exhibiting the most robust production capacity at 581.34 mg/L, which significantly differs from the other four strains (*p* < 0.05). The EPS production of the other four strains were L14 (567.40 mg/L), L5 (546.5 mg/L), L20 (522.21 mg/L) and L17 (450.2 mg/L) respectively. Apart from strain L17 (450.2 mg/L), the remaining four strains all demonstrated EPS production capabilities exceeding 520 mg/L.

#### Adhesion rate to Caco-2 cell line

**Figure 4:**
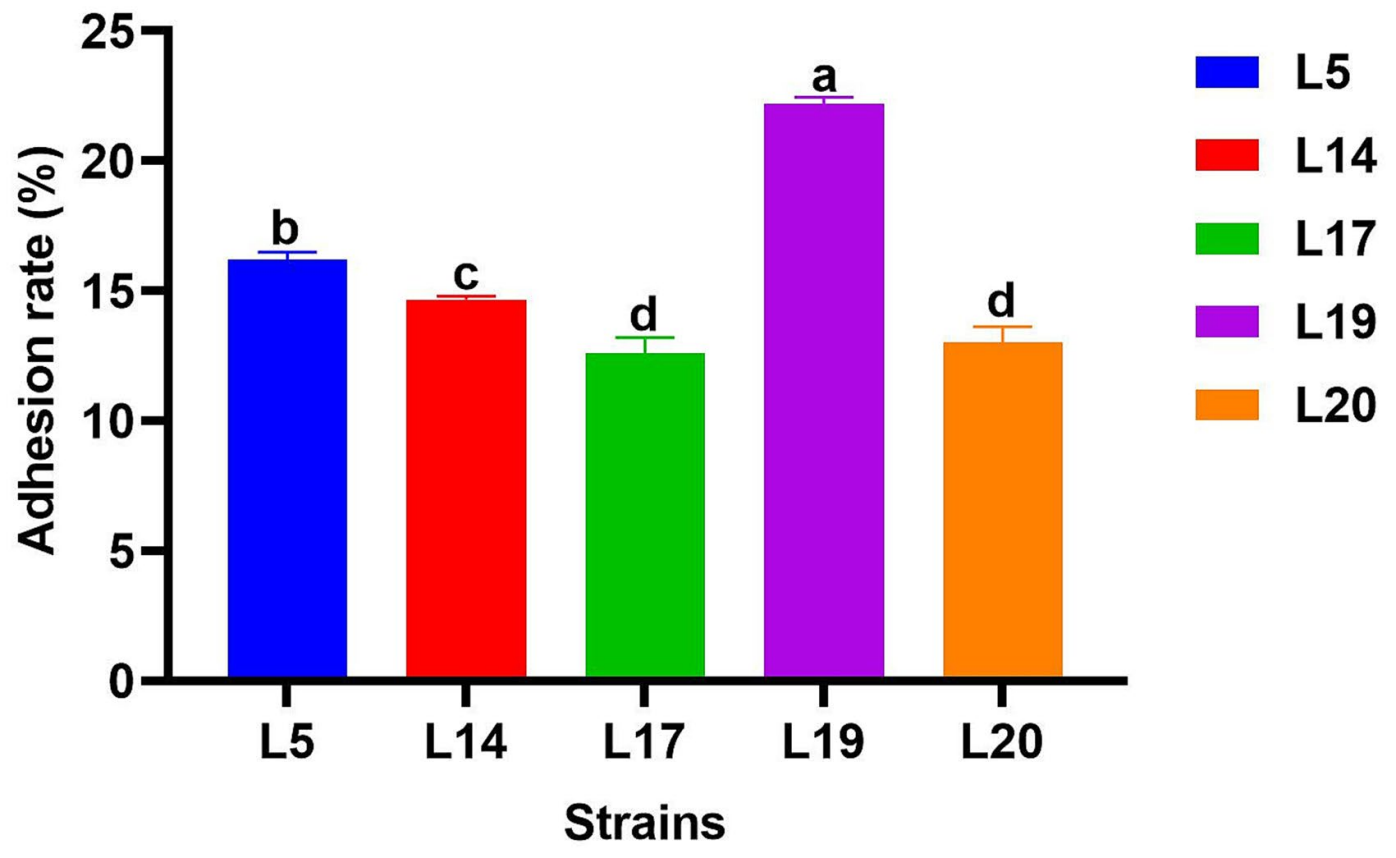
Adhesion rate of LAB to Caco-2 cell line. The data are expressed as mean ± SD. Different letters represent significant difference (*p* < 0.05).

Figure 4 illustrates the adhesion abilities of the LAB to Caco-2 cells. All strains exhibited adhesion to these cells, with adhesion rates varying from 12.6 to 22.19%. Notably, L19 demonstrated the highest adhesion rate at 22.19%, which significantly differs from the other four strains (*p* < 0.05). The adhesion rates of the remaining strains ranked in descending order are L5 (16.20%), L14 (14.33%), L20 (13.01%) and L17 (12.60%).

#### Genomic analysis

Based on the comprehensive evaluation of heat resistance, antioxidant capacity of the five strains, L19 emerged as the most robust and stable in terms of heat resistance and antioxidant properties. Additionally, L19 possesses outstanding adhesion capabilities, holding promise for promising *in vivo* applications. Its exceptional characteristics position it as a promising candidate for the prevention and management of heat stress-related damage. Consequently, the genomic analysis of L19 was conducted, and the findings are presented as follows.

#### Genomic feature

**Figure 5:**
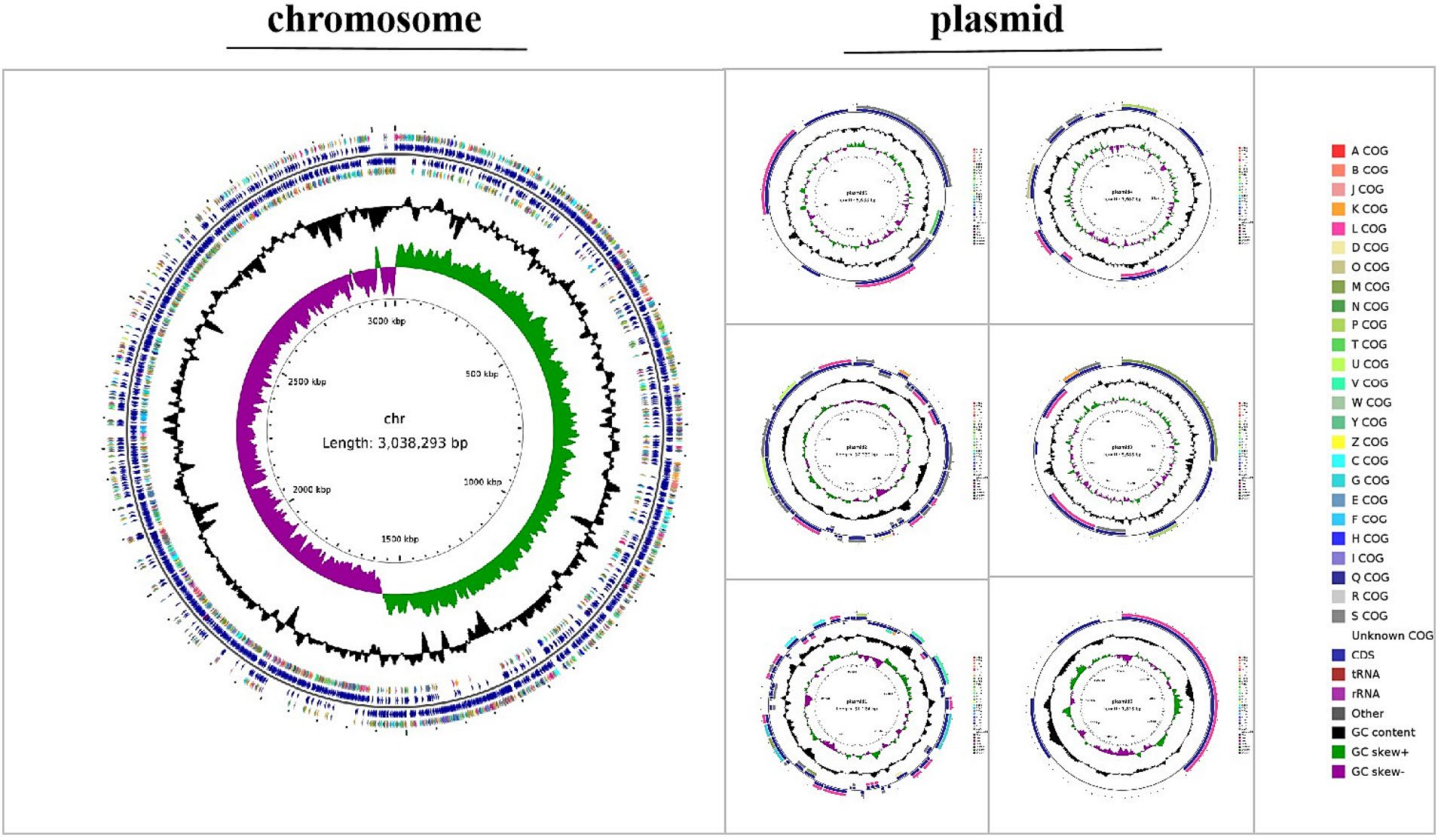
Genomic circle maps of *Lactobacillus plantarum* L19.

The genome of *Lactobacillus plantarum* L19 comprises one chromosome and six plasmids. The total genome size is approximately 3,039,293 base pairs, with a GC content of 44.76%. It encodes a diverse array of RNAs (54 ncRNAs, 67 tRNAs and 16 rRNAs) and harbors one CRISPR sequence. The key genomic features of L19 are summarized in Supplementary Table S1, while the circular genome maps are depicted in Figure 5.

### Functional annotation results

#### COG annotation

**Figure 6:**
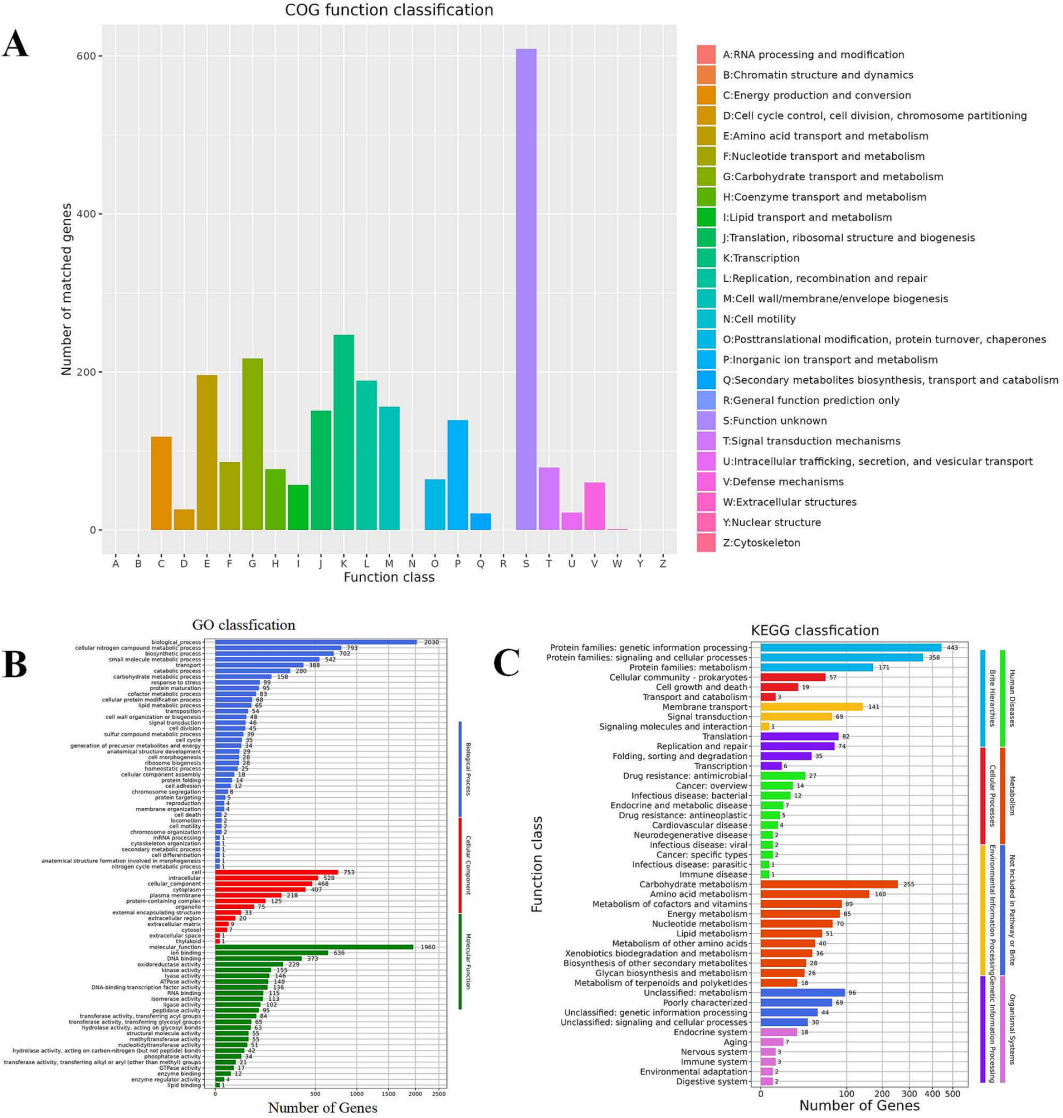
Function annotation results of *Lactobacillus plantarum* L19. **(A)** COG database annotation of *Lactobacillus plantarum* L19; **(B)** GO database annotation of *Lactobacillus plantarum* L19; **(C)** KEGG database annotation of *Lactobacillus plantarum* L19.

Figure 6A reveals that *Lactobacillus plantarum* L19 possesses 19 distinct types of coding genes, as cataloged in the COG database. A detailed examination of Figure 6A indicates that the majority of these genes are primarily associated with the categories of Transcription (247), Carbohydrate transport and metabolism (217), Amino acid transport and metabolism (196), and Replication, recombination, and repair (189).

#### GO annotation

The results of the GO functional annotation for *Lactobacillus plantarum* L19 are depicted in Figure 6B. The GO system categorizes gene functions into three primary domains: Biological Process, Molecular Function, and Cellular Component. The analysis reveals that the majority of genes are primarily involved in cellular nitrogen compound metabolic processes, biosynthetic processes, ion binding, and small molecule metabolic processes. Notably, 229 genes are specifically associated with antioxidant reductase activity, highlighting the strain’s potential in antioxidant-related functions.

#### KEGG annotation

Figure 6C illustrates that *Lactobacillus plantarum* L19 has a total of 1,416 genes annotated in the KEGG database. These genes are categorized into 46 distinct pathways, encompassing eight major functional groups. The most prevalent pathways include Protein families involved in genetic information processing, signaling and cellular processes, and Carbohydrate metabolism. This distribution suggests that L19 possesses robust metabolic capabilities and a strong potential for proliferation. Notably, 85 genes are annotated in the energy metabolism pathway, which is positively correlated with the strain’s antioxidant capacity.

#### Antioxidant-related genes in the genome of *Lactobacillus plantarum* L19

The genome of *Lactobacillus plantarum* L19 was analyzed to identify genes associated with the antioxidant enzyme system, encompassing catalase, superoxide dismutase (SOD), the thioredoxin system, the NADH oxidase-NADH peroxidase system and the glutathione system. As presented in Supplementary Table S2, the L19 genome contains 17 genes related to antioxidant activity.

#### Heat-resistant genes in the genome of *Lactobacillus plantarum* L19

According to the study by Da et al. (Da et al., 2023), as shown in Supplementary Table S3, four genes are intimately associated with the heat resistance of LAB (*adh*E, *his*E, *yku*N and *fol*B). The genome of *Lactobacillus plantarum* L19 harbors 3 out of the 4 heat resistance-related genes (*adh*E, *hid*E, and *fol*B).

#### AMR genes in the genome of *Lactobacillus plantarum* L19

No AMR genes were identified in the genomes of *Lactobacillus plantarum* L19 using ResFinder 4.0.

## Discussion

As global temperatures escalate, the dairy industry faces an increasingly dire challenge from HS, necessitating the urgent development of effective strategies to prevent and mitigate HS-related damages in dairy cows (Ranjitkar et al., 2020). Research shows that enhancing the body’s antioxidant defenses can significantly reduce damage caused by HS. LAB, known for their robust antioxidant properties, safety, and accessibility, have been demonstrated to bolster the body’s antioxidant defenses and counteract oxidative damage (Nakagawa and Miyazaki, 2017), representing a promising intervention for heat stress-related damage in dairy cows. It is widely recognized that probiotics sourced from the same host exhibit greater compatibility with the host, thereby optimizing their probiotic efficacy and enhancing the overall utility of probiotic preparations (Argyri et al., 2013). Therefore, this study aims to screen out heat-resistant and antioxidant strains from cows’ milk to provide effective means and scientific references for preventing and treating of damage caused by HS in dairy cows.

Maintaining superior growth characteristics is crucial for a strain to exert its probiotic properties effectively. Thus, the chosen strain for mitigating HS must withstand high temperatures while retaining growth viability, a factor easily overlooked in strain screening. During HS, body temperature can exceed the normal range, typically reaching 40 ± 0.5°C, which is comparable to the rectal temperature of cows during heat stress (Abeyta et al., 2023; Ebi et al., 2021; Qi et al., 2022). The intestine is the primary site where probiotics exert their beneficial effects. Consequently, in this study, 40°C was used to simulate temperature under HS to evaluate the growth capabilities of LAB under HS. The evaluation results of heat resistance and high temperature of five strains showed L5 and L19 exhibited superior heat resistance and sustained robust growth capabilities under the simulated temperature of HS, which indicates that these two strains can exert good probiotic properties under HS conditions. Furthermore, most probiotic products undergo a spray-drying process (involving high temperatures) during manufacturing, which can significantly impact the survival rate of the strains (Simpson et al., 2005). Therefore, screening strains with excellent thermal stability is also conducive to commercial production.

HS in cows induces oxidative stress through the production of free radicals and peroxides, which is a major cause of oxidative damage caused by HS (Gupta et al., 2023). To combat this, strains with notable antioxidant capabilities are essential. There are various methods to assess probiotics’ *in vitro* antioxidant capacity, including H_2_O_2_ tolerance, reducing and free radical scavenging (DPPH, OH^−^, ABTS) (Zhou et al., 2022). In this study, the antioxidant capacity of five strains was evaluated using the above methods. The results showed that the five strains had different degrees of antioxidant capacity, among which L5 and L19 showed stronger antioxidant capacity, which are exceeded current reports (Li et al., 2012; Liu et al., 2020). Notably, the supernatant’s antioxidant capacity exceeded that of the bacterial suspension, aligning with Wang et al.’s findings (Wang et al., 2023). This indicates that the antioxidant capacity of the supernatant *in vitro* is stronger than that of the bacterial suspension, but the difference in the effects *in vivo* still needs further study. Extracellular polysaccharides (EPS) are natural carbohydrate polymers secreted by LAB during growth. Studies show EPS have antioxidant properties (Wang et al., 2015). This study assessed EPS production in five LAB strains, all capable of producing EPS. The results is in contrast to Espeche et al. (Espeche et al., 2012), in their study none of the LAB isolated from milk produced EPS, possibly due to environmental or strain differences.

For LAB to function as probiotics in the host, their ability to adhere effectively to the intestinal lining is essential (Collado et al., 2006). This study utilized Caco-2 cells to assess the adhesion capabilities of LAB strains. The adhesion rates of the five LAB strains ranged from 12.6 to 22.19%, significantly higher than the 0% reported for *Lactobacillus plantarum* isolated from food by Pinto et al. (Pinto et al., 2020), and the 3.62% for *Lactobacillus plantarum* G83 isolated from panda feces by Wang et al. (Wang et al., 2023). These findings indicate that the LAB strains in this study possess a strong ability to adhere and colonize, which can guarantee its effective probiotic effect in the host body.

Based on a comprehensive evaluation of various attributes, L19 showed excellent heat resistance and antioxidant capabilities, and exhibited superior adhesive capacity and utilization rate, making it a promising candidate for managing the damages of heat stress in cows. To understand its biological role and molecular mechanisms, L19’s genome was sequenced and annotated using GO, KEGG, and COG databases, revealing that L19 possesses a diverse array of antioxidation-related enzymes and energy metabolism pathways, aligning with previous findings (Aziz et al., 2023). Antioxidant enzymes such as superoxide dismutase (SOD) and catalase, the thioredoxin system, the NADH oxidase-NADH peroxidase system, and the glutathione system are collectively recognized as the primary enzymatic defense mechanisms employed by LAB to counteract oxidative stress (Zhou et al., 2022). The genome of L19 was found to harbor 17 genes associated with antioxidant functions, shedding light on its molecular basis for exceptional antioxidant properties. The study of Da et al. on *Lactobacillus plantarum* CGMCC8198 identified *adh*E-like, *his*E, *yku*N, and *fol*B genes as vital for heat resistance, with *his*E being significant. These genes serve as potential markers for heat-tolerant LAB selection (Da et al., 2023). Genomic analysis of L19 confirmed the presence of *adh*E-like, *his*E, and *fol*B genes, aligning with its demonstrated heat tolerance, further emphasizing its potential for withstanding high temperatures. This research represents the first comprehensive analysis of heat-resistant and antioxidant genes in LAB from cow’s milk.

Considering the potential for microbiomes to acquire and transfer antibiotic resistance genes to pathogenic bacteria, posing significant risks (Pinto et al., 2020), the resfinder 4.0 tool was used to analyze the genome of L19 for drug resistance genes (Bortolaia et al., 2020). Fortunately, no drug resistance genes were detected, mitigating concerns about gene transmission contributing to antibiotic resistance. Additionally, the L19 genome sequence was found to contain a CRISPR array, characterized by short, repetitive sequences that are homologous to prophages or plasmids. This feature not only limits the spread of antimicrobial resistance genes but also provides a defense mechanism against incoming extrachromosomal DNA molecules (Marraffini et al., 2006). Overall, these findings confirm the L19 is a safe and reliable strain of LAB.

## Conclusion

In this investigation, a LAB strain (*Lactobacillus plantarum* L19) was screened from the milk of Holstein cows, which exhibited impressive heat resistance and antioxidant capabilities, holding promise for the prevention of heat stress-related damage in dairy cows. The heat resistance and antioxidant capacity of *Lactobacillus plantarum* L19 were verified from the molecular level by genomic analysis, and it did not carry drug resistance genes, ensuring its safety. This study offers a valuable reference and a solid scientific foundation for the application of LAB in preventing and controlling the oxidative damage of HS in dairy cows.

## Data Availability

The data presented in this study can be found in the NCBI database, accession numbers: SRR28342006.
