# Supplementary material for: Screening and genome analysis of heat-resistant and antioxidant lactic acid bacteria from Holstein cow milk
Source: Front Microbiol. 2024 Nov 14;15:1455849. doi: 10.3389/fmicb.2024.1455849 (PMC11602510; doi:10.3389/fmicb.2024.1455849)
Supplement: Supplementary file 1 [file Table_1.DOCX]

Supplementary Material

**Table S1**  The Genomic feature of *Lactobacillus plantarum* L19

| **Features** | ***Lactobacillus plantarum* L19** |
| --- | --- |
| Genominc size (bp) | 3038293bp |
| GC content of chromosome (%) | 44.76bp |
| Gene number | 2902bp |
| Gene total length (bp) | 2535069bp |
| Gene average length (bp) | 873.56bp |
| Gene/ Genome (coding %) | 83.84% |
| Plasmid | 6 |
| ncRNA | 54 |
| tRNA | 67 |
| 5S rRNA | 6 |
| 16S rRNA | 5 |
| 23S rRNA | 5 |
| CRISPR | 1 |

**Table S2** Antioxidant-related genes in *Lactobacillus plantarum* L19 genome

| **ORF_name** | **Gene name** | **Functional annotation** |
| --- | --- | --- |
| chr_2857 | *cat* | catalase |
| chr_1902 | *gsh*A | glutamate--cysteine ligase |
| chr_324、chr_950 | *gor* | glutathione reductase (NADPH) |
| chr_183 | *gpx* | glutathione peroxidase |
| chr_197、chr_1860、chr_2789 | *trx*A | thioredoxin 1 |
| chr_570 | *trx*B | thioredoxin reductase (NADPH) |
| chr_2612 | *trnrd* | thioredoxin reductase (NADPH) |
| chr_49 | *ars*C | arsenate reductase (thioredoxin) |
| chr_1901 | *tpx* | atypical 2-Cys peroxiredoxin |
| chr_1595 | *nox*1 | NADH oxidase (H_2_O_2_-forming) |
| chr_270、chr_818 | *ndh* | NADH dehydrogenase |
| chr_2169 | *zwf* | glucose-6-phosphate 1-dehydrogenase |

**Table S3** Heat-resistant genes of *Lactobacillus plantarum* L19 genome

| **ORF_name** | **Gene name** | **Functional annotation** |
| --- | --- | --- |
| chr_2073 | *his*E | phosphoribosyl-ATP pyrophosphohydrolase |
| chr_2642 | *fol*B | 7,8-dihydroneopterin aldolase/epimerase/oxygenase |
| chr_2884 | *adh*E | alcohol dehydrogenase |
